# Epidemiological study of snakebite cases in Sikkim: Risk modeling with regard to the habitat suitability of common venomous snakes

**DOI:** 10.1371/journal.pntd.0009800

**Published:** 2021-11-08

**Authors:** Ananta Rai, Manita Chettri, Sailendra Dewan, Bidita Khandelwal, Basundhara Chettri

**Affiliations:** 1 Department of Zoology, School of Life Sciences, Sikkim University, Tadong, Gangtok, East Sikkim, India; 2 Department of General Medicine, Sikkim Manipal Institute of Medical Sciences, Sikkim Manipal University, Gangtok, East Sikkim, India; Fundação de Medicina Tropical Doutor Heitor Vieira Dourado, BRAZIL

## Abstract

**Background:**

Snakebite envenoming is listed as category ‘A’ Neglected Tropical Disease. To achieve the target of WHO (World Health Organization) 2019, it becomes necessary to understand various attributes associated with snakebite including community awareness, improvisation of medical facilities and to map the potential distribution of venomous snakes responsible for the bite. Hence this study is conducted in Sikkim, India to understand the epidemiology of snakebite in Sikkim. The potential distribution and risk mapping of five common venomous snakes are done for effective management of snakebite cases.

**Methods and findings:**

The snakebite cases registered in six district hospitals and four PHCs (Primary Health Centers) of Sikkim were collected from the year 2011 to 2018. Community survey was also conducted to supplement the data. Ecological Niche Modeling (ENM) was performed to predict the potential habitat of five common venomous snakes of Sikkim. The risk modeling of snakebite cases was done at the level of Gram Panchayat Unit (GPU) using Geographically Weighted Regression (GWR) and Ordinary Linear Square (OLS) model. We found higher number of male victims inflicted with snakebite envenomation. The potential distribution of the five venomous snakes showed satisfactory mean AUC (Area under Curve) value. Both the models showed significant positive association of snakebite cases with habitat suitability of the venomous snakes. Hospital data revealed no death cases whereas community data reported 24 deaths.

**Conclusions:**

Death from snakebite reflected in community data but not in hospital data strongly indicates the people’s belief in traditional medicine. Though people of Sikkim have rich traditional knowledge, in case of snakebite traditional practices may be ineffective leading to loss of life. Sensitizing people and improving medical facilities along with proper transport facilities in rural areas might significantly reduce the snakebite casualties in the state.

## Author summary

Snakebite is a global burden especially inflicting despair and torment among agronomist rural population of the society. This study has been conducted in small state of Sikkim in India which has not been actually considered so far in any of the national statistical condensation of snakebite cases. There are around 19 venomous snakes found in Sikkim of which five common ones co-exist with humans. Such co-existence may lead to unintentional encounter posing life threatening snakebite. People of Sikkim have strong belief on traditional healers for treatment of snakebite which results in mortality and morbidity. The present epidemiological study provides the baseline information compiling both hospital and community data and predict the areas having higher risk of snakebite envenomation in Sikkim. This study will help the authorities for better preparedness to deal with snakebite emergency to achieve minimum or no mortality. Potential risk mapping will help the health department for stocking anti-venom in nearby health centers. Most importantly, this study will create awareness among the people and the authority about snakebite which is considered as neglected tropical disease.

## Introduction

Snakebite is one of the prominent health hazards in tropical countries and is recognized as Neglected Tropical Diseases (NTD) by World Health Organization (WHO) in 2009 [1]. This was followed by World Health Assembly (2018) and its main aim was the development of global strategy to decrease the number of snakebite envenomation and death by 50% by 2030 [2]. According to WHO, about 5.4 million people of the world is at risk of trespassing venomous snakes. Approximately 7400 people are envenomed everyday while about 81,000–138,000 people die annually by snakebite and more than the double number around 400,000 people are left with physically and physiologically disarmed annually [2,3].

The Indian statistical scenario on mortality and envenomation by snakebite has remained imprecise since long. Annual snakebite death has been estimated to be approximately 50,000 cases [4] including 61,507 bites with 1124 deaths in 2006 and 76,948 bites with 1359 deaths in 2007 [5]. The MDS (Million Death Study) reported total national snakebite death of 2833 from the year 2001 to 2014 with 27 deaths from Assam alone and 37 from other North-eastern states (Arunachal Pradesh‚ Manipur‚ Meghalaya‚ Mizoram‚ Nagaland‚ Sikkim and Tripura).Total snakebite death was estimated to be about 808,000 from 2001 to 2014 by applying weighted 3-yearly moving average method to MDS data and the UN Population Division. The systematic review of 78 literatures from 24 states published over 20 years reported 87,590 snakebite cases (both fatal and non-fatal) from 2000 to 2019 out of which 3329 were death cases [6]. Further, Salve et al. (2020) [7] reported a total of 200,492 envenomed cases due to snakebite in the year 2017–18 and 230,950 in the year 2018–19 from India. Despite acceleration of studies in the recent years, the pragmatic scenario of snakebite in India is still not absolute because the regional or state level studies are very scanty.

Sikkim although placed under lower burden states, lackfield based studies on snakebite epidemiology. Based on the data available on online portal, Salve et al. (2020) [7] reported 15.3 and 14.4 persons with snakebite envenomation from Sikkim per 100,000 populations in the year 2017–18 and 2018–19 respectively. However, most of the Indian snakebite studies are based on hospital records [5,7–9] which do not reflect the true scenario, as many victims visit traditional healers, while many die even before reaching the hospital. Thus, community based epidemiological study becomes necessary to understand the actual rates of snakebites and deaths [10,11]. Therefore, this study aims to understand the epidemiology of snakebite and its associated factors in Sikkim by compiling both hospital and community based data. The study also attempts to predict the high risk area which will help in minimizing the conflict with snake in Sikkim.

Sikkim is home to about 71 species of snakes [12] among which only 19 species are venomous. Species such as *Naja kaouthia*, *Ophiophagus hannah*, *Bungarus niger*, *Ovophis monticola* and *Protobothrops himalayanus* are commonly encountered venomous snakes of Sikkim. Although these venomous snakes are listed as category 1 and 2 of venomous snakes by WHO, *Ovophis monticola* and *Protobothrops himalayanus* are not included [13]. The commonness of these species is based on the frequency of sightings. Known the fact that snakes inhabit varied domains, sometimes encompassing human habitation, the risk posed by snakes, especially venomous snakes is quite consequential in public health [4]. Moreover, venom composition vary between and within snake species, hence the design and manufacture of species specific antivenin requires information on the diversity and distribution of venomous snakes and their toxins, which is not available in most countries at present [14]. Thus, it becomes pivotal to understand the potential distribution of venomous snakes to anticipate risk of snakebite associated with the area. Therefore, we used Ecological Niche Modeling (ENM) to understand the potential distribution of the venomous snakes of Sikkim based on bioclimatic variables, slope, aspect, Land Use and Land Cover (LULC), Normalized Difference Vegetation Index (NDVI) and distances to water sources. Additionally, Ordinary Least Square (OLS) and Geographically Weighted Regression (GWR) were used to understand the spatial relationship between snakebite cases and ecological factors associated with habitat suitability of venomous snakes and also with socio-economic and demographic variables. The area associated with snakebite risk was mapped at the Gram Panchayat Unit (GPU) level. This holistic approach is vital in addressing the ongoing global snakebite crisis at the regional level.

## Materials and methods

### Ethics statement

Ethical clearance for the study was obtained from the ethical committee of Sikkim Manipal Institute of Medical Sciences, 5th Mile, Tadong, Gangtok, Sikkim– 737102; dated 7^th^ September 2019 with the approval number–SMIMS/IEC/2019-110. Aim of the study was explained to all the respondents and verbal consent was obtained prior to the interview. Youngest respondents were fifteen years old and the questionaire was asked to him/her after obtaing verbal permission from their parents.

### Study area

The study was conducted in the state of Sikkim (27° 5’ - 28° 9’ N and 87° 59’ - 88° 56’ E) in India which is a part of Himalayan Biodiversity Hotspot [15] and covers geographical area of 7096 km^2^. Sikkim shares its boundaries with three countries namely, Bhutan in the East, Nepal in the West and Tibetan Autonomous Republic of China in the North and North East. The average annual rainfall ranges from 1300 mm at the lower valleys to 4300 mm at the mountain ridges and around 60–70% of the rainfall occurs during the monsoon season i.e. June to September [16]. The vegetation of Sikkim is categorized into three major types–Tropical, Temperate and Alpine [17]. According to 2011 census, the total population of Sikkim is 610,577 and the density is 86 persons per sq km. Administratively, Sikkim is divided into four districts, 40 blocks and 185 Gram Panchayat Units (GPUs). GPU is a basic village level unit of Indian administration used as clusters consisting of village or few villages divided into wards (www.desmesikkim.nic.in).

There are four district hospitals located at Singtam, Namchi, Gyalsing and Mangan and two multispecialty hospitals namely, Sir ThutobNamgyal Memorial (STNM) hospital, Gangtok and Central Referral Hospital, Gangtok. There are 24 PHCs (Primary Health Centers) in Sikkim with six in East, seven in West, six in South and five in North district (https://www.sikkim.gov.in/departments/health-family-welfare-department).

### Data collection

#### Snakebite data based on hospital and community survey

Data on snakebite cases of Sikkim were obtained through two methods; (i) cases registered in the hospitals and PHCs, and (ii) community-based survey. For the hospital based data collection, all the four district hospitals, STNM hospital and the Central Referral Hospital (CRH) were considered and data were extracted from the year 2011 to 2018. Since North, West and South Sikkim projected comparatively inadequate data, two PHCs from North district (Chungthang and Lingthem), one from West district (Dentam) and one from South district (Temi) were also considered. There are three hospitals in East Sikkim, hence no PHCs from East district were considered for data collection. Data were collected with the prior permission from Medical Superintendent and Commissioner-cum-Secretary of Health and Family Welfare Department, Government of Sikkim. Our work has been approved by the ethical committee of CRH, Gangtok (Registration No. SMIMS/IEC/2019-110 dated 7^th^ September 2019). Cases that had been referred from the PHCs to the district hospitals were also noted for subsequent follow up in the respective hospitals. During the data collection, each hospital (including PHCs) was visited and information such as date of admission and discharge, reason of admission, name, sex, age and address of the patient were recorded. This snakebite data were also segregated according to GPU to use as a variable in GWR and OLS modeling. However, due to lack of trained health care workers and insufficient information provided by the victim, the species responsible for the bite could not be anticipated. Information on the symptoms of venomous bite was also unavailable in the hospital record.

Community-based survey was conducted from June 2019 to December 2019 in all the four districts of Sikkim (four villages were randomly selected from each district) using pre-designed questionnaire method. From each village, ten respondents were randomly selected (regardless of whether they are victim of snakebite or not). Predesigned questionnaire was made and a prior verbal consent of each respondent was obtained before the interview. The respondents were interviewed based on structured questionnaire covering aspects such as age, occupation, qualification, personal snakebite experience, symptoms and activity during the bite, whether they had witness snakebite cases or deaths in their community, common habitat of snake, time and season of encountering snakes if any and their perception on snakes (S1 Text). Respondent’s feedback was noted and the figures were converted to percentage. Repetitive information on snakebite cases and deaths from the villages were verified and noted to avoid ambiguity in the data.

#### Socio-demographic and land use land cover data

Since snakebite is associated with human population density and their activities, total population of different villages, forest area, net crop sown area, etc. at the GPU level of Sikkim were obtained from the Department of Economics, Statistics, Monitoring and Evaluation (DESME), Government of Sikkim, India. The data were based on the census of India, 2011. We used all these variables for OLS and GWR.

#### Ecological Niche Modeling (ENM) of venomous snakes

The occurrence records of five most common venomous snakes of Sikkim used in ENM model namely *Ovophis monticola* (Mountain Pit Viper), *Protobothrops himalayanus* (Himalayan Pit-Viper), *Naja kaouthia* (Monocled Cobra), *Ophiophagus hannah* (King Cobra) and *Bungarus niger* (Greater Black Krait) were obtained through field surveys, opportunistic sightings and one record of *Ovophis monticola* from Global Biodiversity Information Facility (GBIF; http://www.gbif.org). During field survey, coordinates and elevations of the location were recorded using Garmin78S GPS. These five snake species were selected considering their commonness and wide range of distribution in Sikkim. Total of 14 presence records were obtained for *O*. *monticola*, eight records for *P*. *himalayanus*, 11 records for *N*. *kaouthia*, nine records for *B*. *niger* and nine records for *O*. *hannah* (S1 Table). These presence record data were used to model the distribution of these species across Sikkim.

CHELSA (Climatologies at high resolution for the earth’s land surface areas) environmental dataset [18] were used as predictor variables for species distribution modeling. CHELSA is a high resolution (30 arc sec) climate data set for the earth land surface areas consisting of 19 bioclimatic variables derived from the monthly mean, maximum, minimum temperature, and mean precipitation values. They represent annual trends (e.g., mean annual temperature, annual precipitation), seasonality (e.g., annual range in temperature and precipitation) and extreme or limiting environmental factors (e.g. temperature of the coldest and warmest month, and precipitation of the wet and dry quarters). The datasets were downloaded from https://envicloud.wsl.ch/#/?prefix=chelsa%2Fchelsa_V1%2Fclimatologies.

Apart from bioclimatic variables, slope, aspect, LULC, NDVI and distance to water courses were also considered for distribution model building. Digital Elevation Model (DEM) imagery (covering the Sikkim Himalayan region) was downloaded and generated from the Cartosat-1 satellite, built and operated by the Indian Space Research Organization (ISRO). DEM was used to compute slope and aspect using spatial analysis tool in ArcGIS v10.4. Furthermore, since hydrology also plays a significant role in shaping the habitat of species, the thematic layer “distance to water sources” was computed using Euclidean distance tool in ArcGIS v10.4. Landsat cloud-free time-series imagery were selected for preparing decadal LULC layers [19].

The Normalized Difference Vegetation Index (NDVI) was also used as one of the variables for building the model. The three years (2016–2018) of Landsat8 imagery data (available at 30 m resolution) for the Sikkim Himalayan region was used which is available from the USGS3 website. The three years of red and near-infrared imagery data was averaged and then NDVI was calculated from these averaged outputs using the formula NDVI = (Near Infrared–Red) / (Near Infrared + Red) in ArcGis10.4. The final output provides NDVI values for each pixel.

To account for multicollinearity between the set of 19 bioclimatic variables, elevation, slope, aspect, LULC, NDVI and distance from water sources, correlation analysis was performed using ENM Tools 1.3 and redundant variables with high correlation (r > 0.9 and <-0.9) were not included in species distribution modeling [20]. The six bioclimatic variables retained from CHELSA were Bio 1 (Annual Mean Temperature), Bio 2 (Mean Diurnal Range), Bio 3 (Isothermality), Bio 12 (Annual Precipitation), Bio 14 (Precipitation of Driest Month) and Bio 19 (Precipitation of Coldest Quarter).

MaxEnt (Maximum entropy modeling of geographic distribution) version 3.4.4 [21] was used to estimate the potential distribution of the venomous snakes of Sikkim. The distribution model was generated using the occurrence record, six bioclimatic CHELSA variables, slope, aspect, LULC, NDVI and distance to water sources. Ten replicated models were executed for the species to validate the model robustness with 10 percentile training presence logistic threshold [22]. Quality of the model was evaluated based on AUC value [23], a value above 0.9 indicates strong prediction ability, values between 0.7 to 0.9 indicate moderate ability and values below 0.7 indicate poor prediction ability [24]. The habitat suitability areas of the species were mapped using ArcGIS by importing the averaged probability model output. The predicted probability distribution was grouped into five suitability classes viz., very low (0–0.2), low (0.2–0.4), medium (0.4–0.6), high (0.6–0.8) and very high (0.8–1.0).

### Data analysis

Explorative data analysis was performed with hospital and community based data. The number of cases obtained from the hospital survey was segregated based on age group, sex and district and was assessed using χ^2^ test. Community data were segregated based on their response to the structured questionnaire. The number of respondents was categorized based on age group, sex, occupation and educational qualification.

Geographically Weighted Regression (GWR) model in combination with Ordinary Least Square (OLS) model was used to access the relationship between snakebite and explanatory variables such as human population records, probability of snake occurrence in the area, forest area and net area of cultivated land at GPU level. The habitat suitability value generated from ENM that lied within the boundary of each GPU were extracted and averaged. This average value for each of the venomous snakes was used as one of the predictor variable in GWR. OLS is a commonly used regression methods to analyze the relationship between a set of parameters. It is a global regression statistics that assumes relationship between the parameters which remains constant over space and does not vary (homoscedasticity). The other assumption of OLS is that the model residuals should not be spatially autocorrelated. However, the spatially structured dataset often violates these assumptions and may therefore hinder the results. Therefore, GWR statistics are often used to compensate for both non-homoscedasticity and spatial autocorrelation in a dataset [25,26]. GWR is a type of local regression statistics that models the relationships between parameters by constructing a separate OLS regression for each sampling points. The GWR uses a distance decay approach for weighting all the observations within a kernel bandwidth around given sampling point [27]. Thus, the observations closer to the sampling points will have greater influence on estimation of parameters. The GWR uses a kernel bandwidth size as a threshold to specify the rate of influence of coefficients with increasing distance from the sampling points [28]. We compared both the OLS and GWR to explore which regression models performed best to explain the snakebite cases in Sikkim. Among the two regression models, one with highest coefficient of determination (R^2^) and lower AIC was chosen as the best fit model to predict snakebite incidence. Both the analyses were performed in Multiscale Geographically Weighted Regression (MGWR v 2.2.1) software.

## Results

### Hospital records

#### Snakebite cases among different age-groups

**Fig 1:**
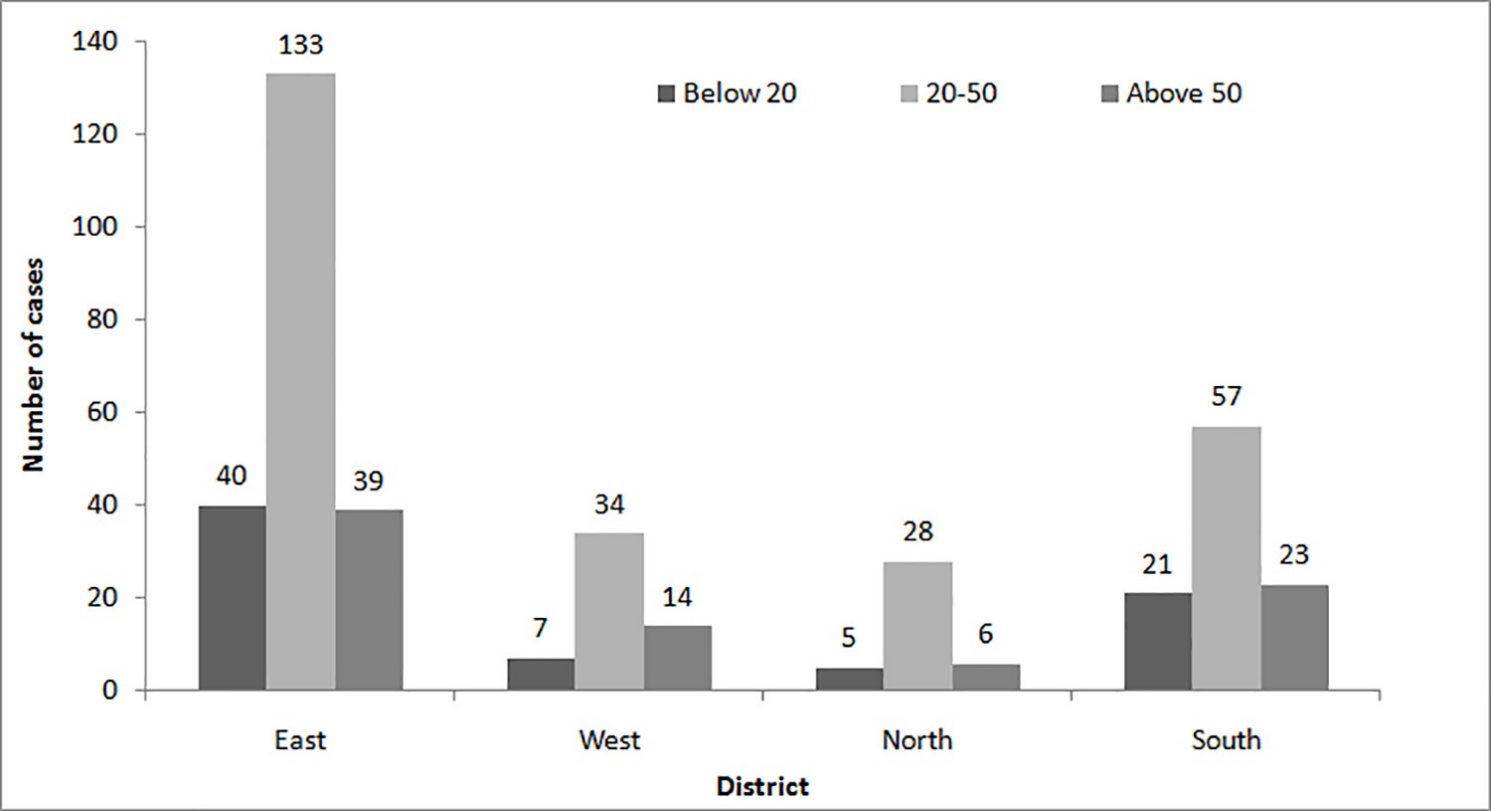
Distribution of snakebite cases among populations of different age-groups in four districts of Sikkim.

During 2011–2018, a total of 409 cases were registered in six hospitals and four PHCs of Sikkim. Out of 409 cases, 247 (~60%) were males and 162 (~40%) females; male victims were significantly higher than the females (Fisher exact test; p = 0.0001, df = 1). No death cases were registered in hospitals as well as PHCs. Snakebite cases seemed to be highest from the age group between 20–50 years in all the four districts of Sikkim. North district recorded lowest cases of snakebite i.e. 39 which accounts for 0.089% of the population (43,709) whereas East district recorded highest with 214 cases representing 0.075%of the population (283,583). The other two districts South and West recorded 101 (0.068%) and 55 (0.04%) cases with apopulation of 146,850 and 136,435 respectively (Fig 1).

#### Monthly and yearly distribution of snakebite cases

**Fig 2:**
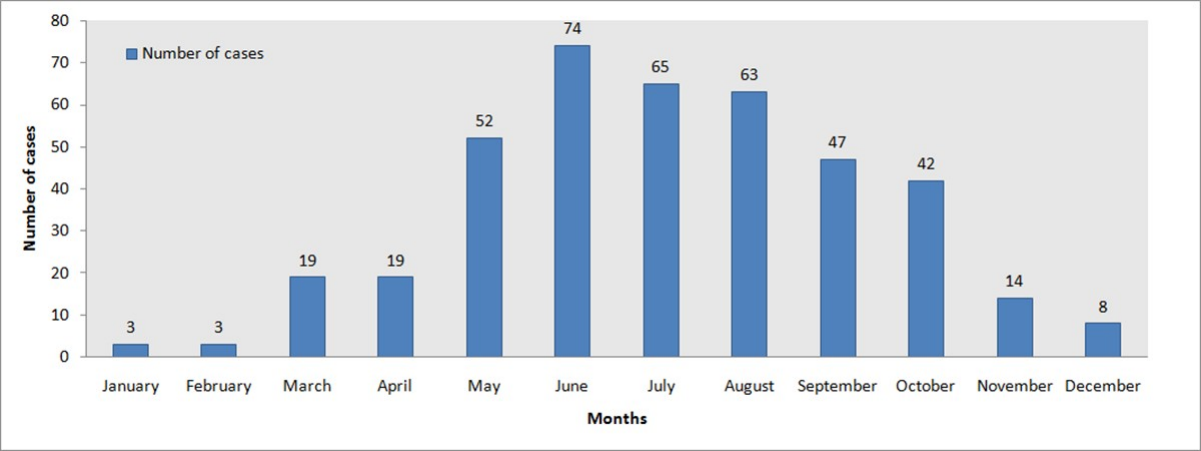
Monthly distribution of snakebite cases based on hospital record of Sikkim from 2011 to 2018.

Snakebite cases followed unimodal pattern of seasonality with highest incidence (254 cases ~ 62%) during monsoon from May to August while the incidence were comparatively lower during pre-monsoon (January to April) and post-monsoon (September to December) seasons (Fig 2). Yearly snakebite incidence showed increasing trend from 2011–2018. Out of the total 409 cases, maximum snakebite cases i.e. 117 (~28%) was observed in the year 2018 followed by the year 2016 with 93 (~23%) cases and the least was in 2011 with 11 (~2.61%) cases.

### Community based-survey

#### Socio-demographic characteristics of respondents

In the community based survey, total of 160 respondents were interviewed based on the structured questionnaire, out of which 90 (~56%) were males and 70 (~44%) were females (Table 1). Out of 160 respondents, more than 60% were from age group of 20–50 years. Majority of respondents (>60%) were either illiterates or had primary level education. Predominant proportions of respondents were farmers (~59%).

**Table 1:** Socio-demographic characteristics of the respondents (n = 160) from the community survey in Sikkim Himalaya, India.

Respondent variable	Sub-group variables	Number of respondents (%)
Gender	Male	90 (56)
	Female	70 (44)
Age category	Below 20 years	20 (12.5)
	20–50 years	100 (62.5)
	Above 50 years	40 (25)
Educational Qualification	No formal education	43 (27)
	Basic primary education	61 (38)
	Secondary education	22 (14)
	Higher secondary education	18 (11)
	Higher education (12^th^ and above)	16 (10)
Employment	Farmer	95 (59)
	Self employed	27 (17)
	Government employee	24 (15)
	Students	14 (9)

#### Personal encounter of snakes and witnessing snakebite at the community level

Out of the total 160 respondents, 150 (~94%) respondents had encountered snake during their lifetime and hence were exposed to snakebite risk. Most of the encounters were during day time between 10am to 3pm (~68%) while relatively low frequency of encounters were in the morning (~8%), evening (~13%) or night time (~10%). Highest encounters were during monsoon (~70%) and low in other seasons (pre-monsoon ~10% and post monsoon ~19%). Predominant encounters were in the farmland (~40%) while performing agricultural activities. Among 150 respondents who had encountered snakes, 89 (~54%) heard of snakebite cases in their community. The number of cases reported by each respondent varied from 1–6 accounting for 212 snakebite cases and 24 death cases in the study area since 2005. This makes total of 236 snakebite cases obtained through community based survey (Table 2). Similar to hospital data, the relative percentage of snakebite cases is found to be highest in North (0.118%) followed by South (0.066%) and lowest in East (0.017%) district with respect to population of the district.

**Table 2:** Snakebite morbidity and mortality data based on community survey from four administrative districts of Sikkim, India.

District	Snakebite cases	Snakebite death	Total
East	45	4	49
West	32	5	37
North	51	1	52
South	84	14	98
**Total**	**212**	**24**	**236**

#### Snakebite prevalence based on personal snakebite experience and people’s perception on snakes

Of the total 160 respondents, 89 respondents who had witnessed snakebite in their community, 28 (~31.4%) respondents themselves were victims of snakebite. Of the 28 personal cases, 25 did not visit hospital for treatment and were treated by traditional healers while rest three cases were taken to hospital. Of the total snakebite cases, almost 28% were unreported in the hospital data. There were 16 (~ 57%) male victims and 12 (~43%) female victims. Most of the respondents (~51%) projected general fear towards snake while few (~21%) felt that snakes play an important role in maintenance of ecological balance and believed it to be of religious significance, and some (~15%) found it useless in nature while others (~13%) had a neutral feeling towards snakes.

#### Elevational range of commonly encountered five different venomous snakes of Sikkim

**Fig 3:**
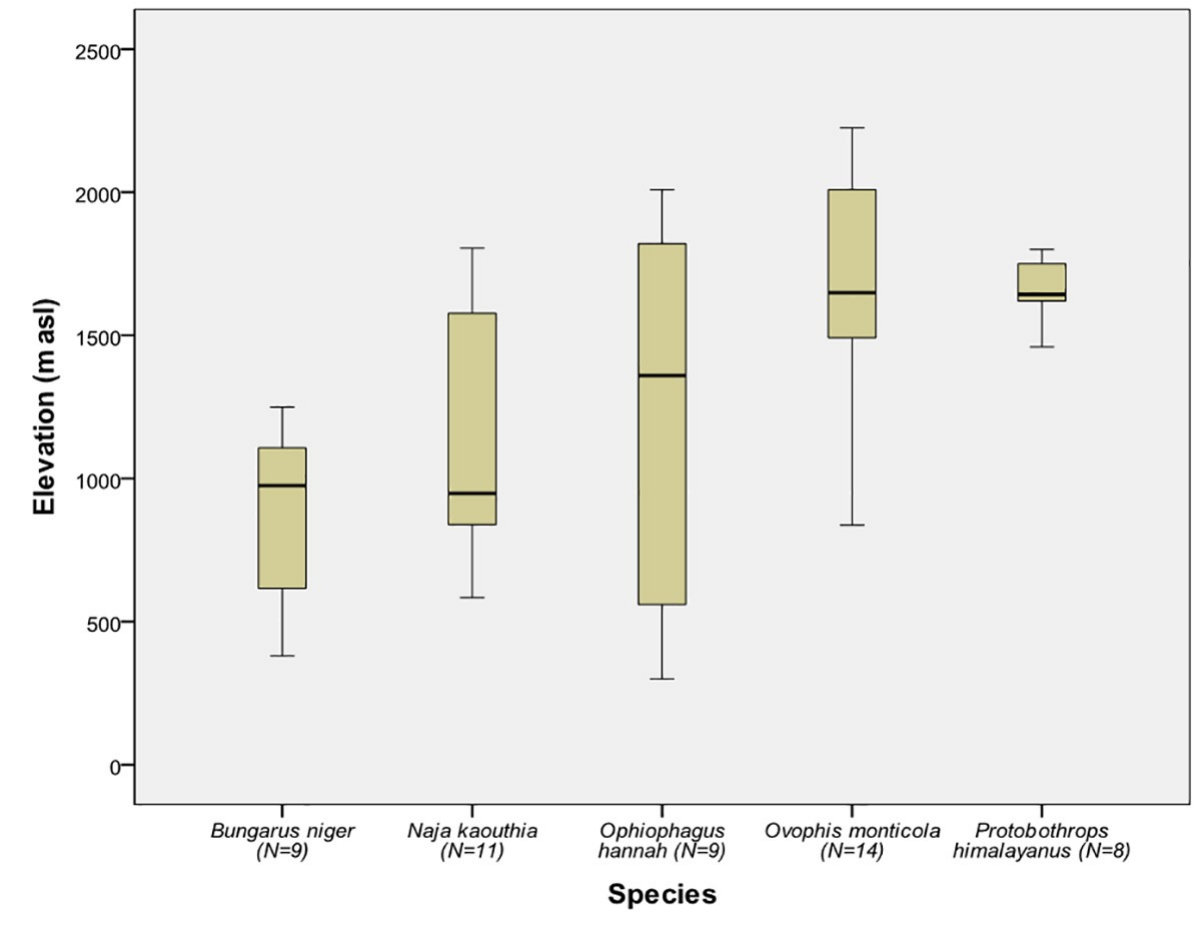
Elevational distribution range of five common venomous snake of Sikkim (Thick line indicates the mean and box represent theelevationalrange of each species along with the standard error.

The elevation range varied between different venomous snakes of Sikkim (Fig 3). *Bungarus niger* were distributed along the elevation range of 450m to 1300m while *Naja kaouthia* were found between 500m to 1800m. *Ophiophagus hannah* showed wide range of distribution from 300m to 2000m. Two species of pit-vipers, *Ovophis monticola* and *Protobothrops himalayanus* are found along the mid-elevation zone between 800m to 2500m and 800m to 2100m respectively. Most of the venomous snakes were distributed from low to mid-elevation in Sikkim.

### Ecological Niche Modeling

**Fig 4:**
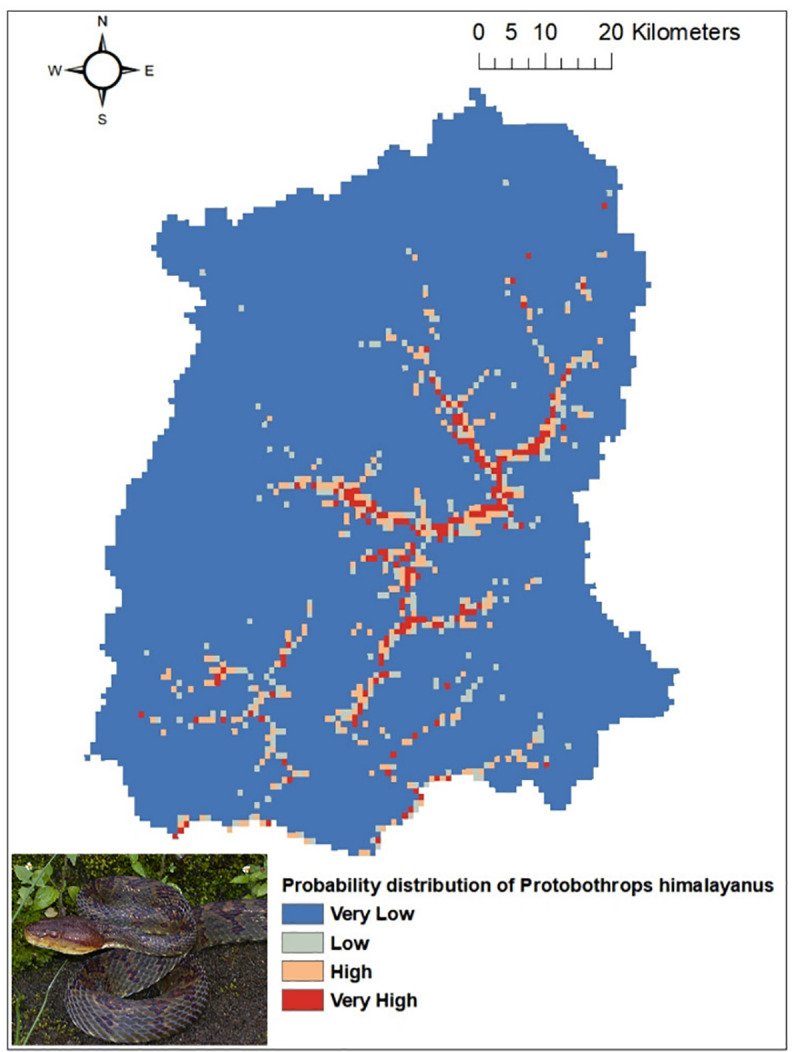
Potential distribution of *Protobothrops himalayanus* (Himalayan pit viper) in Sikkim as predicted by ENM.

**Fig 5:**
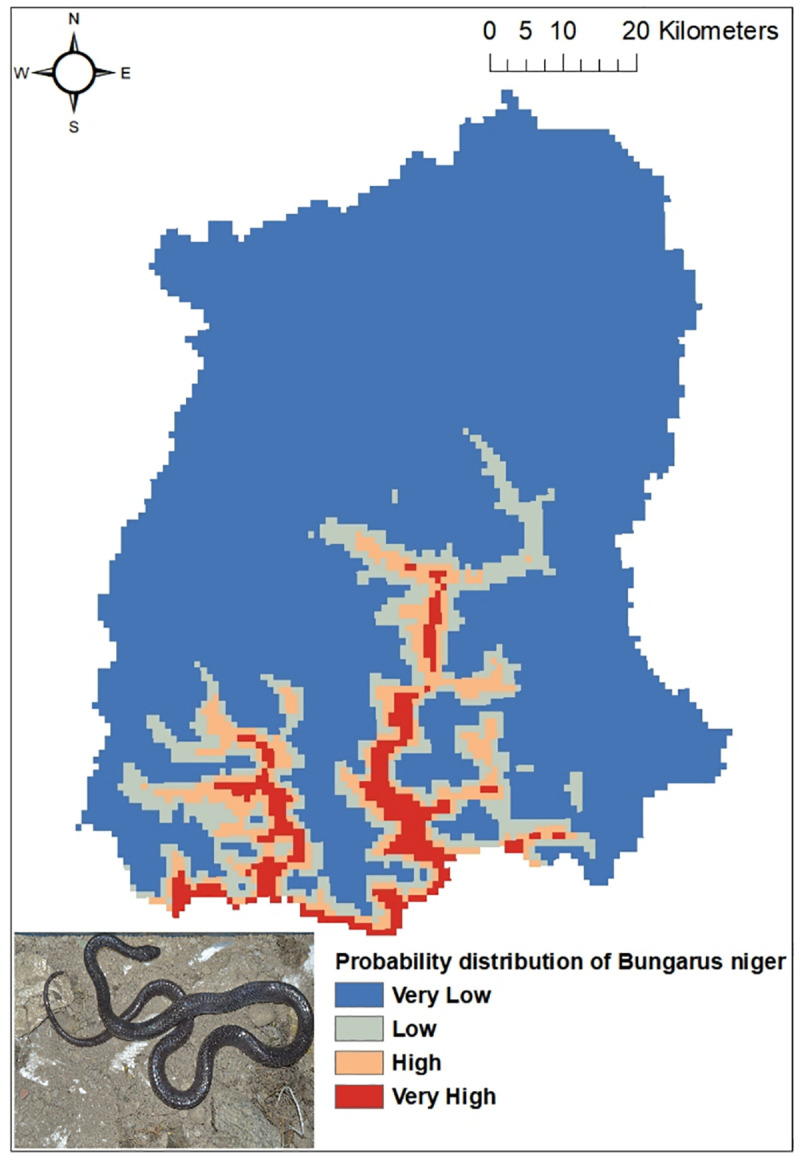
Potential distribution of *Bungarus niger* (Greater black krait) in Sikkim as predicted by ENM.

**Fig 6:**
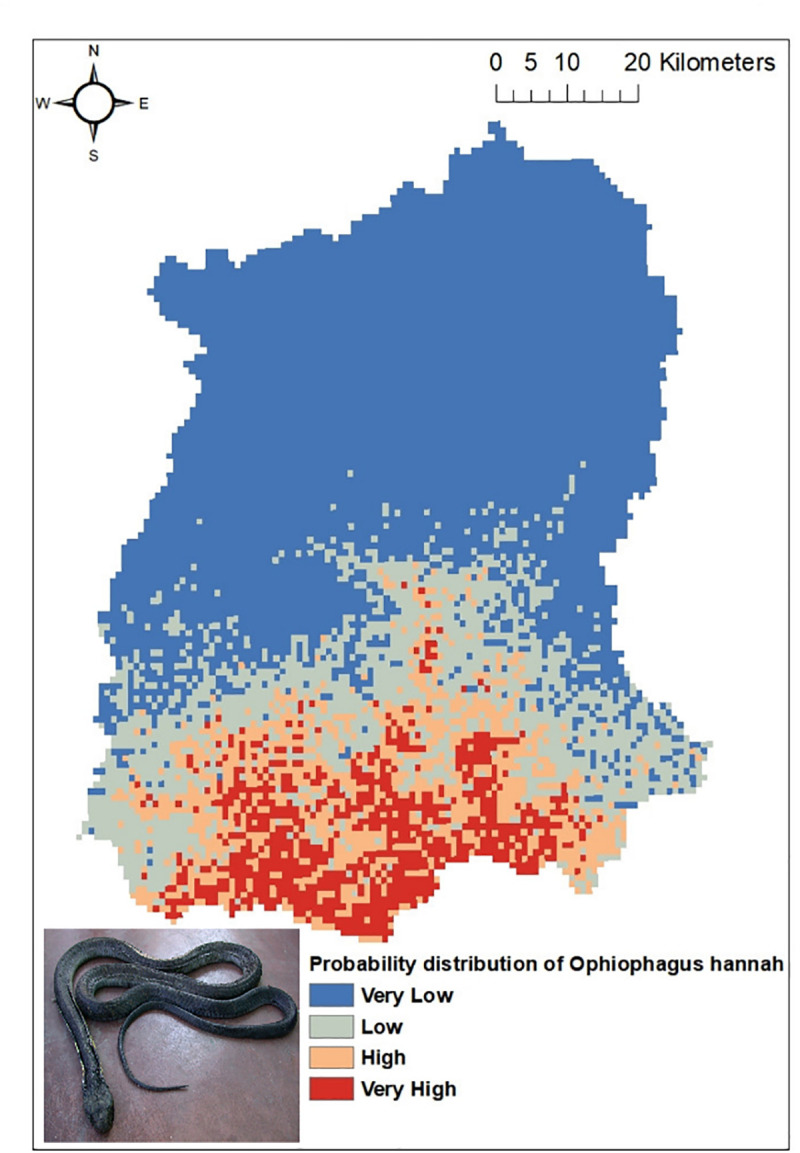
Potential distribution of *Ophiophagus hannah* (King Cobra) in Sikkim as predicted by ENM.

**Fig 7:**
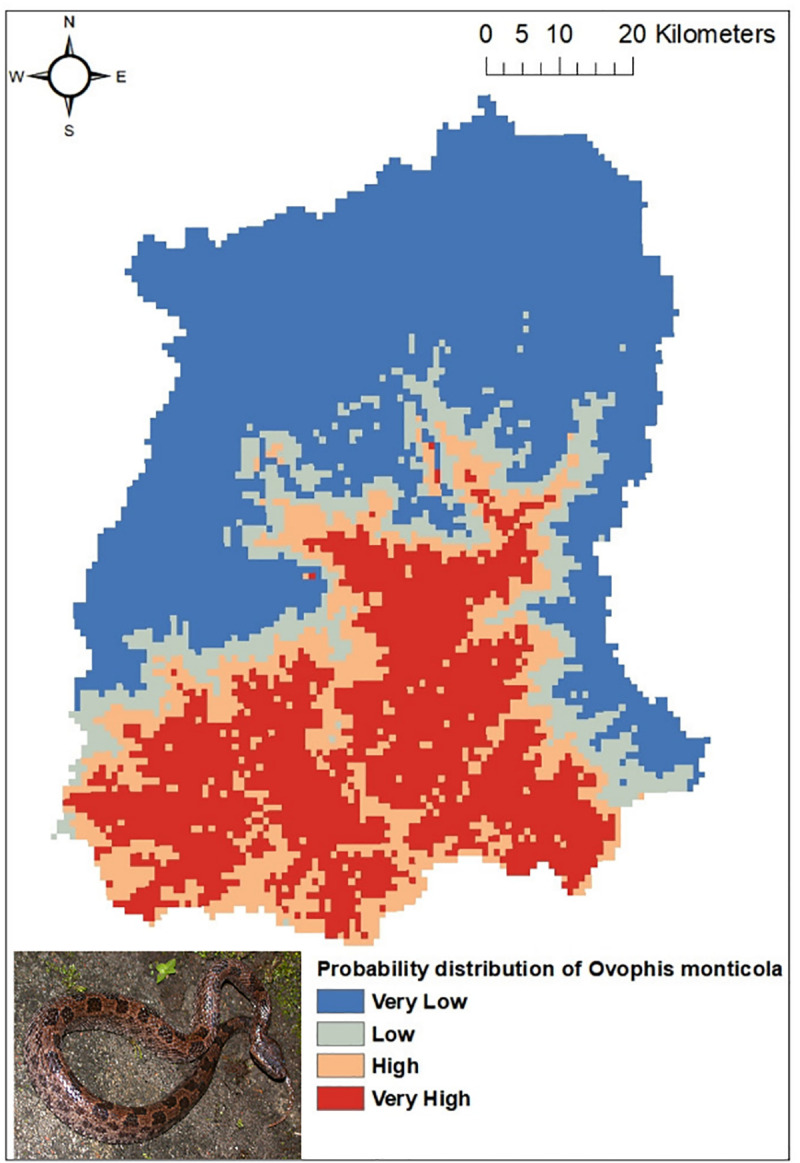
Potential distribution of *Ovophis monticola* (Mountain pit viper) in Sikkim as predicted by ENM.

**Fig 8:**
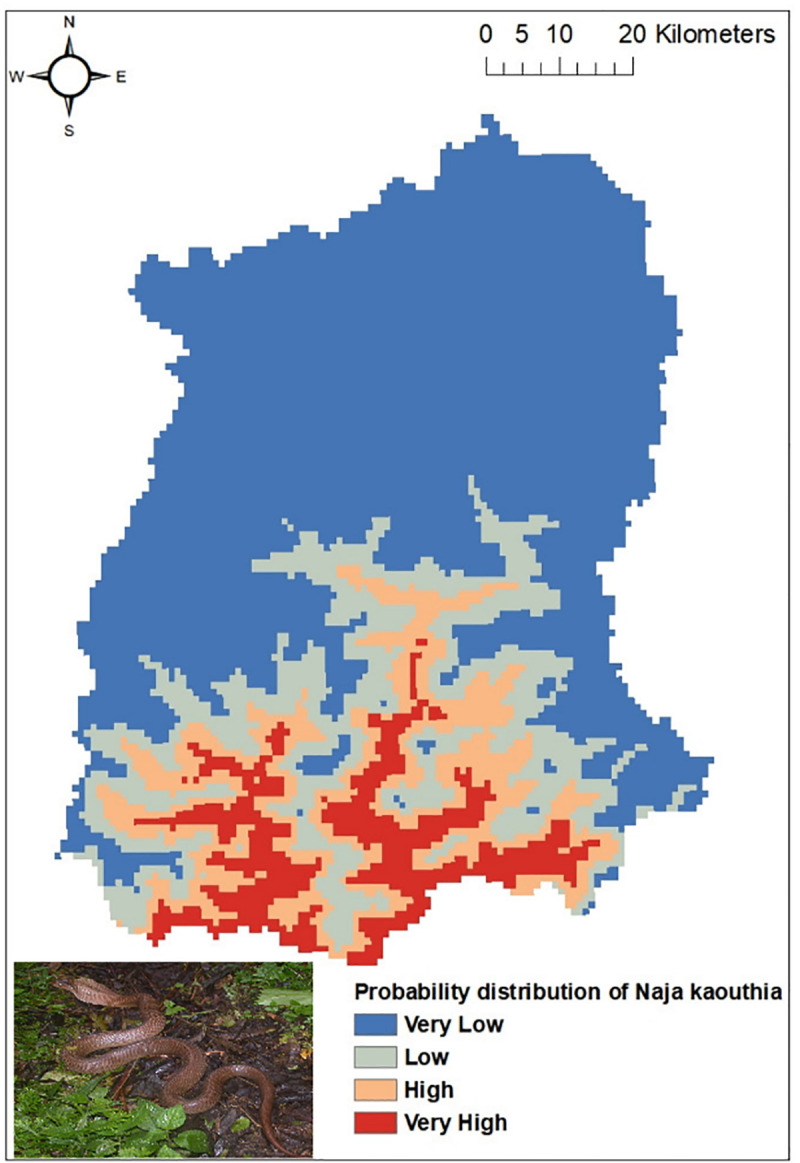
Potential distribution of *Naja kaouthia* (Monocled Cobra) in Sikkim as predicted by ENM.

**Fig 9:**
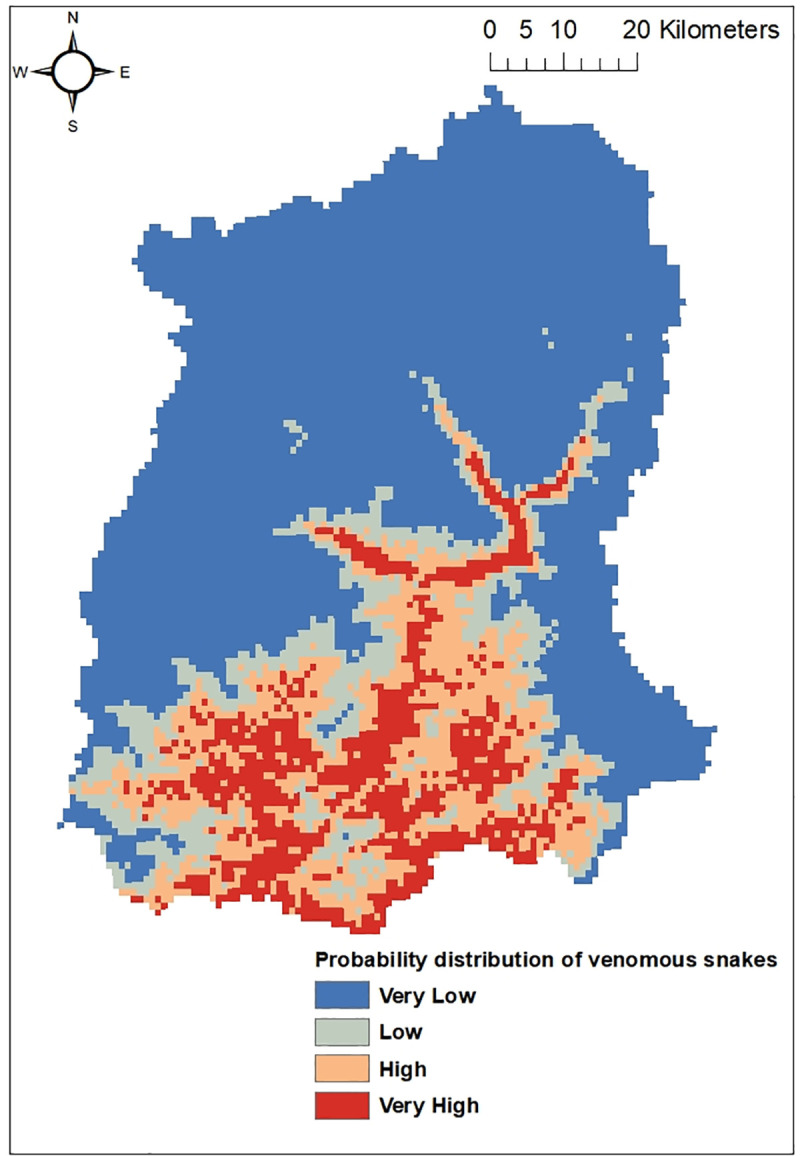
Potential distribution of common venomous snakes of Sikkim (all five together) as predicted by ENM.

Out of the five common venomous snakes, the potential distribution for the *Protobothrops himalayanus* (Fig 4), *Bungarus niger* (Fig 5) and *Ophiophagus hannah* (Fig 6) developed by MaxEnt model have shown good prediction with mean AUC value of 0.903 (±0.186), 0.960 (±0.030), and 0.900 (±0.061) respectively. However, potential distribution model have given moderate prediction for *Ovophis monticola* (Fig 7) and *Naja kaouthia* (Fig 8) with mean AUC value of 0.769 (±0.100) and 0.889 (±0.070). The model for the overall venomous snakes (all the five venomous snakes together; Fig 9) gave a moderate prediction with AUC value of 0.868 (±0.038).

Among all the environmental variables used for building the model, percentage contribution of AMT (Bio 1) was highest for all the venomous snakes except *Ophiophagus hannah*. In case of *Protobothrops himalayanus* five variables (AMT, AP, Aspect, Distance to water and PCQ) contributed almost equally accounting for 93.7% while for *Ovophis monticola*, *Bungarus niger* and *Naja kaouthia* the highest contribution was by AMTwith percentage contribution of 82.9, 80.6 and 80.1 respectively. For *Ophiophagus hannah*, mean diurnal range has highest contribution in distribution model with percentage contribution of 41.7 followed by LULC with 26.8%. The details regarding the percentage contribution of different environmental variables in habitat suitability model of different species is given in Table 3.

**Table 3:** Percentage contribution of different environmental variables for building habitat suitability model usingMaxEnt for five common venomous snakes of Sikkim along with the number of presence records and AUC value (figures in bold represent two highest contributing factors).

Species	Number of presence records	Environmental variables and their percentage contribution (%)											AUC (mean ± SD)
		Bio 1 (AMT^a^)	Bio 2 (MDR^b^)	Bio 3 (I^c^)	Bio12 (AP^d^)	Bio 14 (PDM^e^)	Bio19 (PCQ^f^)	Slope	Aspect	LULC^g^	NDVI^h^	DW^i^	
***Protobothropshimalayanus***	8	**21.3**	0	0.3	**19.4**	0	16.5	0.7	18.8	9	5.2	17.7	0.903 ± 0.186
***Ovophis monticola***	14	**82.9**	**5.9**	0	0.5	0	0	1.1	1.7	0.1	7.1	0.6	0.769 ± 0.10
***Bungarus niger***	9	**80.6**	1	0.1	1	3.1	**12.8**	0	0.7	0.2	0	0.5	0.960 ± 0.030
***Naja kaouthia***	11	**80.1**	**14.2**	0	1.1	0	0	0.2	0	0.6	0	3.7	0.889 ± 0.070
***Ophiophagushannah***	9	22.8	**41.7**	0	0	0	0.4	7.5	0.7	**26.8**	0.1	0	0.90 ±0.061
**All five together**	51	**83.9**	1.1	2.9	**5.9**	0.4	0.7	0.5	0.8	3.5	0.2	0.3	0.868 ± 0.038

^a^AMT-Annual Mean Temperature

^b^MDR- Mean Diurnal Range

^c^I-Isothermality

^d^AP-Annual Precipitation

^e^PDM- Precipitation of Driest Month

^f^PCQ- Precipitation of Coldest Quarter

^g^LULC- Land Use and Land Cover

^h^NDVI- Normalized Difference Vegetation Index

^i^DW–Distance to water sources.

### Snakebite risk mapping

The OLS model produced an R^2^ value of 0.086 and R^2^adj value of 0.068 with an AIC of 447.693 and an AICc of 450.082. Output from the GWR model using a fixed Gaussian kernel with a bandwidth of 160 km resulted in an R^2^ of 0.326and an R^2^adj value of 0.225 with an an AIC of 434.353 and an AICc of 441.546 (Table 4).

**Table 4:** Value of evaluation criteria for OLS Regression and GWR model.

	R2	Adj R2	AIC	AICc
**OLS Regression**	0.086	0.068	447.693	450.082
**GWR**	0.326	0.225	434.353	441.546

The AICc value for the GWR model was lower than that of the OLS model. Therefore, the GWR model was considered to be the best fit model. However, both OLS and GWR showed positive association of venomous snakes with habitat suitability hence both models were considered (Table 5).

**Table 5:** p-value of different variables for OLS Regression and GWR model.

	OLS regression model	GWR model
Variables	p-value	p-value
Intercept	0.4601	1.000
Habitat suitability	**0.001**	**0.0103**
Population	0.226	0.300
Forest area	0.252	0.770
Net cultivated Area	0.182	0.197

**Fig 10:**
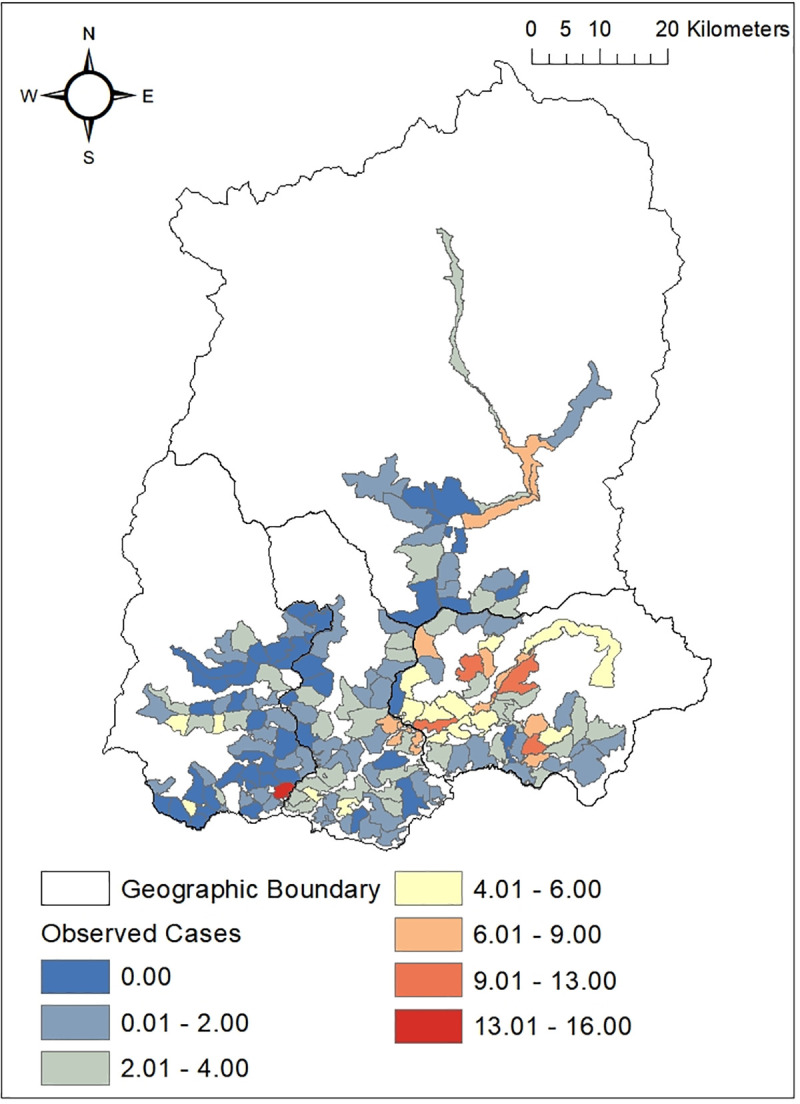
Distribution of snakebite cases in different Gram Panchayat Units (GPUs) of Sikkim, India.

**Fig 11:**
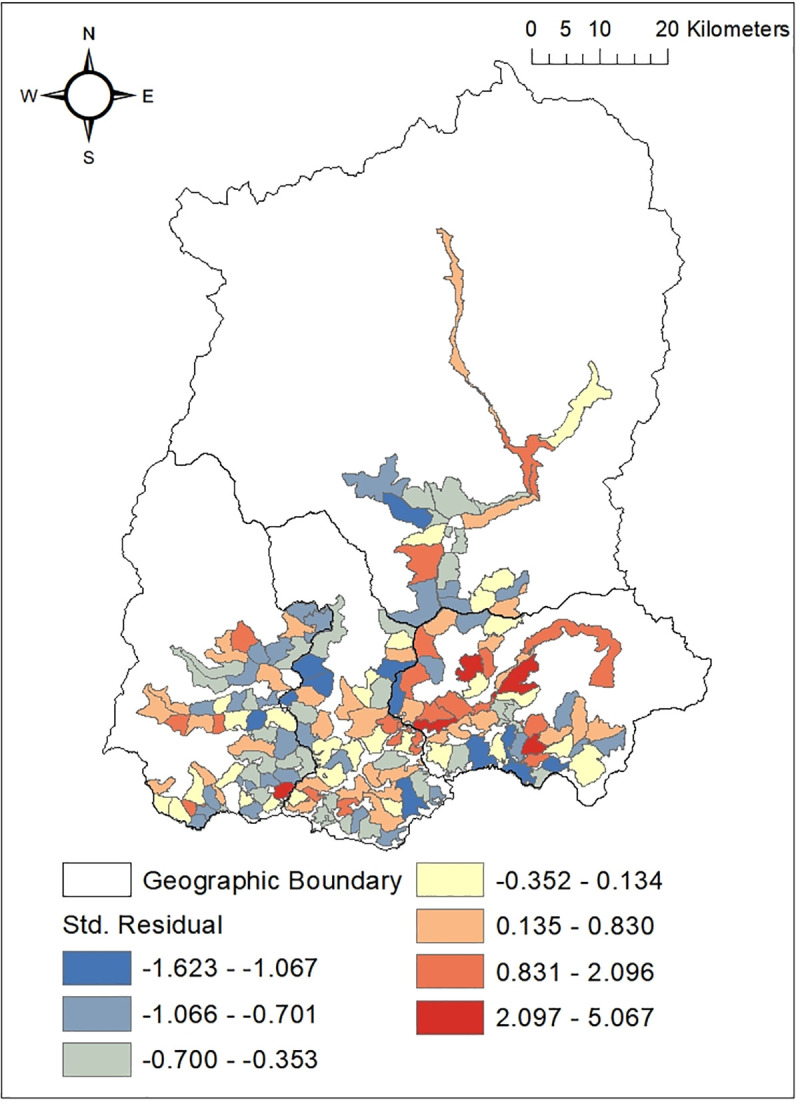
Mapping of snakebite risk based on the GWR (Geographically Weighted Regression) residual value.

The mapping of the snakebite cases in GPU (Gram Panchayat Unit) of Sikkim and mapping of the GWR residual value was done using ArcGIS v10.4 (Figs 10 and 11). The residual with negative values in the map indicates overprediction in area with lower cases of snakebite. This probably stipulates a missing explanatory variable in the model. The value was high in four GPUs of East Sikkim namely Bering Tarethang, Rey Mendu, Sirwani Chisopani, SamdongKambal and one GPU of West Sikkim i.e.Samsing Gelling.

## Discussion

Understanding the potential risk of snakebite is a local, national and global concern to achieve the target of WHO to reduce the number of snakebite associated deaths and disabilities to half by the year 2030. WHO has set combination of approaches such as empowerment and engagement of community, improved access to modern health facilities and enhanced cooperation among stakeholders at various levels. Although conflicts with snakes result in loss of life and permanent disabilities more than the cumulative loss caused by other wildlife, snakebite has always been neglected. Here, we have studied the snakebite cases and its associated factors and identified the potential risk area.

The snakebite cases (hospital as well as community based data) showed higher incidences in males than in females. Majority of the rural population in Sikkim are involved in cattle rearing especially cows and goats which requires collection of fodders, cutting of grasses, etc. Since male individuals are engaged in outdoor activities, they are more prone to snakebite compared to females. Also, economically productive age group (21–50 years) are more vulnerable to snakebite contributing 60% cases. This is in line with other studies conducted in India [4], Bhutan [29], Brazil [30] and Northern Ghana [31]. This pushes the entire family into poverty as the victims belong to economically productive age groups who are the bread earners [14]. Government of India has passed a bill “Payment of Compensation to Victims of Natural Calamities and Snake Bite Act, 2014” to compensate the victims belonging to three different categories-minimum payment of 200,000 INR in case of death, 50,000 INR in case of bodily harm and insurance scheme for persons residing near areas frequented by venomous snakes. However, people residing in rural areas are not aware about this bill. Hence, sensitizing people about the government compensation is necessary so that exacerbate economic burden to the family can be reduced. Based on hospital data, East district of Sikkim recorded highest number of cases and North district recorded lowest. Highest record in East district is due to referred cases from other districts to STNM and CRH hospital which are hospitals with basic facilities to deal with snakebite emergency situation. However, relative percentage of cases with respect to population was found to be highest in North district and least in West district (0.04%).Similarly, community survey also revealed highest percentage of cases with respect to population in North district though the number of cases was highest in South district. Both hospital and community data showed highest percentage of snakebite cases in North Sikkim. Hence, there is a need to improvise the health and transport facilities in North district to deal with snakebite emergency. North district includes some of the remote and isolated areas of Sikkim which leads to difficulty in accessing the medical facilities of hospitals. North Sikkim is geographically and geologically difficult and there is lack of good emergency transport system [32]. Further, North-east India has been projected as vulnerable area based on the duration to reach urban health centers which are equipped to treat envenomation cases [33]. Along with access to safe and effective healthcare, transport and communication infrastructure has been listed as obstacles by WHOto reduce and control snakebite envenomation.

Snakebite incidences were more frequent during monsoon i.e. from June to September which is directly proportional to the breeding period of snakes when they are highly active in search of food as well as mate [31]. Monsoon is characterized by ideal temperature and humidity favorable for snakes and also for the people to carry out their agricultural activities thus increasing the conflict. Similar observation was made by previous studies in Nepal and Bhutan [29,34]. During winters (December to February) temperature being low, snakes go for hibernation [29] which results in very few or no cases of snakebite. Such data will be helpful for better preparedness to reduce the snakebite burden. Yearly increasing trend of snakebite incidence from 2011-2018 is most likely due to improvement in record keeping in the recent years.

Community based data revealed that most victims (89%) visited traditional healers which signifies people’s faith in the traditional method of treatment. Recently on 31^st^ July 2021, a young boy of 13 year old lost in his life due to envenomation of cobra (as per victim’s description) in Yangang, South Sikkim. The victim was taken to traditional healers first and when the condition deteriorated further, he was taken to hospital almost after 8 hours of bite. Injection of eight vials of polyvalent ASVcould not save the victims. Sooner the victims receive medical treatment, greater is the chances of recovery and survival [2]. The community has strong faith on traditional healers which results in delayed or no treatment thus increasing the chances of morbidity and mortality.

**Fig 12:**
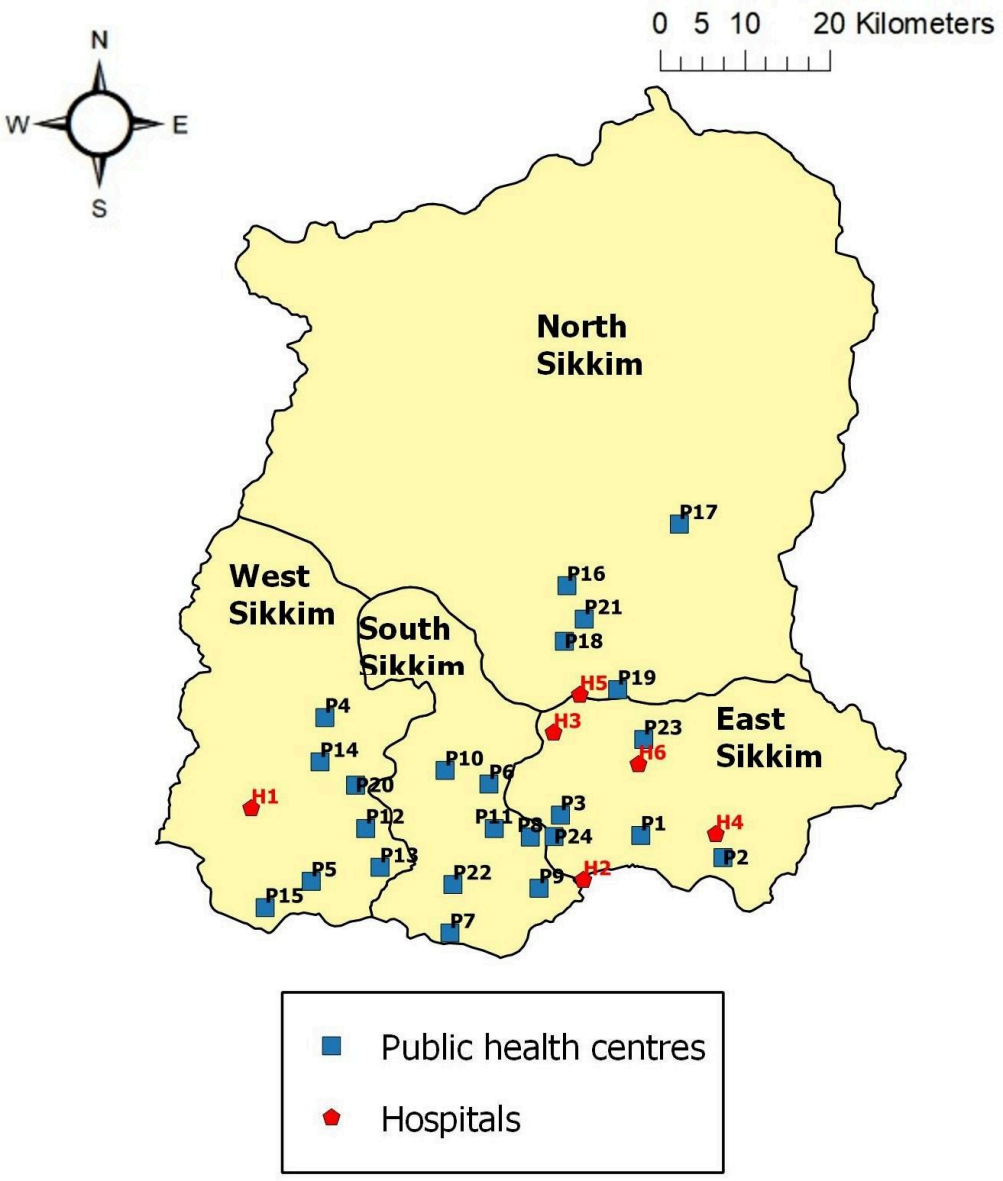
Location of hospitals and PHCs (Primary Health Centers) in Sikkim, India. **[Primary Health Centres** [P1—Passingdang PHC; P2—Chungthang PHC; P3—Dikchu PHC; P4—Hee-Gyathang PHC; P5—Phodong PHC; P6—Rangpo PHC; P7—Samdong PHC; P8—Machong PHC; P9—Pakyong PHC; P10—Rongli PHC; P11—Sang PHC; P12—Yangang PHC; P13 –Melli PHC; P14—TokalBermoik PHC; P15—Namthang PHC; P16—Ravangla PHC; P17—Temi PHC; P18—Yuksom PHC; P19—Soreng PHC; P20—Dentam PHC; P21—Rinchenpong PHC; P22—Mangalbaria PHC; P23—Singyang PHC; P24—Sombaria PHC]. **Hospitals** [H1—Mangan district hospital; H2—Multispeciality hospital, Sikkim; H3—Singtamdistrict hospital; H4—Central referral hospital, Manipal; H5—District hospital, Namchi; H6—Gyalsing district hospital].

While six hospitals and 24 primary health centers of Sikkim may cater to approximately 0.6 million populations of Sikkim (one PHC for 20,000–30,000 populations, as per Govt. of India rule), the availability of polyvalent antivenom and the associated medical facilities required for snake bite emergency are not ensured in all health centers. Further, most of the PHCs are clustered and are not uniformly spread throughout geographical area (Fig 12). Hence, their accessibility in terms of distance, road and transport during snakebite emergency has to be reassessed.

Data from hospitals recorded no death cases but community survey revealed 24 death cases. Therefore, hospital data was supplemented with community survey to get clearer picture of snakebite in Sikkim. Though, the sample size was small from community survey, it gives insight into one of the important issue of public health for the first time in the region. Majority of inhabitants of Sikkim are indigenous tribes who depend on their rich ethnozoological knowledge for the treatment of various ailments including snakebite [35]. The faith that pervades in the rural society of Sikkim regarding the traditional healers is so intense that people prefer visiting them especially in case of snakebite rather than opting modern medical facility. Similar finding was reported from Srilanka with 43.3% victims seeking traditional treatment for snakebite [36]. Raising awareness and educating people about identification of snakes, their ecology and effectiveness of modern medicine might motivate them to visit hospital rather than traditional healers.

Majority of the respondents were farmers who are directly associated with the habitat of snakes on a regular basis and they were either illiterates or just had basic primary education. Previous study also suggested that snakebite envenoming must be treated as a major health issue since it disproportionately affects the lower socioeconomic segment of the society with people having poor housing, limited access to education and health care leading to deprivation, despair and loss of income in the family [14,31]. Majority of the respondents had encountered snakes during their life time and the encounter was often during daytime in monsoon. Agricultural activities, collection of firewood and fodder etc are regular activities of rural areas of Sikkim. This leads to unintentional encounter of snakes during their outdoor activities increasing the risk of snakebite. Taking certain precautions while working in agricultural field, cardamom plantation and grass cutting such as wearing gloves and boots, avoid putting bare hands in tall grasses or stone piles, walking with torch light and stick during night might reduce conflict with snakes.

Snakes occupy wide range of habitat from forest, villages, farmlands and also in urban spaces, hence it is inevitable to avoid human-snake interaction. Further, the general prejudices that prevail regarding snakes in human society cannot be overlooked as they prove to be vital from conservation perspective [37]. Majority of the respondents expressed general fear from snakes and they often tend to prosecute snakes considering all the snakes to be fatal to humans. Rat snakes and cobras, the common snakes encountered near human habitation, compete strongly for food and resources, thus avoid each other. Hence, sensitizing people to coexist with the non-venomous rat snakes might save them from the deadly cobra (DrPratyushMohapatra, *pers*. *comm*.). It is important to sensitize people that not all snakes are venomous and understanding the ecology of individual snakes helps in better management of the venomous snakes.

Understanding snakebite risk and its prevalence pertains to several interrelated factors concerned with both snake and human. Therefore, it becomes important to understand the density, activity pattern and behavior of the snake as well as that of its victim [31]. The species responsible for the bite could not be determined due to lack of systematic records and trained personnel for snake identification. The victims were also unable to identify snakes responsible for the bite, hence after showing symptoms they were treated with Snake Antivenin (Polyvalent) I.P. as per the severity of bite. Though, there are five species of venomous snakes which are common in Sikkim, the presence of any one venomous snake makes the community vulnerable. Different species contribute differently to envenomation risk, species with high incidence of envenomation might be the reason for high snakebite burden of the locality [33]. Eight individuals of Greater Black Krait (*Bungarusniger*) was opportunistically sighted in human habitation in and around Gangtok (East Sikkim) between 2018 and 2019 indicating their higher abundance near human habitation in Sikkim. Similarly, cobras were often encountered along the village trails and roadside during day time and were found entering the cowshed or poultry coops and even human houses during night. Pit-vipers are also common in Sikkim and they often take refuge in cardamom plantations and agriculture field in search of rodent prey. The anti-venin specific to these snakes have not been produced so far and the hospitals in Sikkim are still supplied with the polyvalent antivenin used against “The Big Four” venomous snakes of India. The effectiveness of these anti-venin against the species found in Sikkim is yet to be ascertained. Since species responsible for the bite and their specific symptoms were not available in hospital and community data, we could not identify the snake having higher snakebite burden.

However, considering the wide distribution and common encountering of the five venomous snakes of Sikkim, the potential habitat model was built using the Ecological Niche Modeling utilizing the occurrence record of the snakes, the bioclimatic and environmental variables. Based on MaxEnt model, both temperature and precipitation influences the distribution of *Protobothrops himalayanus* while temperature alone contributed significantly in the distribution of *Ovophis monticola*. Both species of viper is found in middle elevation between 1000-2500m where the precipitation is maximum (2500-3300mm) and the temperature is moderate (16–23°C). This elevation zone is also suitable for large cardamom plantation. The plantation requires shade tree which keeps the area moist and cool and also removal of unwanted weeds and grasses from cardamom plantation during monsoon season provides thick leaf litter bedding on the floor. The cool temperature and moist leaf litter bedding provides ideal habitat for pit-vipers. Similarly, AMT contributed maximum in the distribution of *Bungarus niger* and *Naja kaouthia* and mean diurnal range for *Ophiophagus hannah*. These species are mostly distributed within an elevation range of 200-1000m in Sikkim. Since they are mostly low elevation species, temperature is key factor for limiting their distribution. However, there is a report of *O*. *hannah* from 1820m from Yuksom, West Sikkim [38] which could be indication of elevational range shift.

The habitat suitability model predicts that all the four districts have suitable habitat for *Ovophis monticola*. Areas in South and East districts are predicted to have suitable habitat for *Bungarus niger*, whereas *Naja kaouthia* and *Ophiophagus hannah* has its high probability of distribution towards the lower elevation area of East, West and South districts of Sikkim. Through citizen science, the occurrence of *Ophiophagus hannah* was noticed in Rhenock, Rongli, Central Pandem and Rangpo in East Sikkim, Tharpu in West Sikkim and Namchi, Kamrang and Kitam in South Sikkim (S1 Table). The risk of snakebite in a particular geographic area is substantially determined by the distribution of venomous snakes of an area and other variables such as demographic factors, activities of the inhabitant of that area and environmental variables leading to human snake interaction [36].

Snakebite risk mapping using the GWR residual value predicted four GPUs of East district—Bering Tareythang, Rey Mendu, Sirwani Chisopani, Samdong Kambal and one GPU of West (Samsing Gelling) to be at highest risk of snakebite. These areas cover the lower elevation belt of Sikkim with comparatively higher annual mean temperature making the areas suitable for *Bungarus niger*, *Naja kaouthia* and *Ophiophagus hannah* and therefore increasing the risk of bite. These areas where snakebite risk has been highlighted can be surveyed to determine the true incidence of envenoming. It is recommended that the primary health centers and the district hospitals located in these GPUs should have regular stock of snake anti-venin along with necessary equipments and properly functioning transport system to promptly deal with emergency situation arising out of human snake conflict. While the present study provide insights on snake bite cases and associated risk, more extensive studies with long term data is necessary to have more understanding on the issues for execution of proper management and planning.

## Conclusion

The target of WHO to reduce the snakebite to half by 2030 is a very promising approach to deal with human snake conflict. While cumulative loss due to snakebite surpass other wildlife conflict, the studies on epidemiology of snakebite remains fairly understudied. In India, an average of 50,000 snakebite cases and envenomation is reported every year. Sikkim, though listed in lower burden states, there are ample cases of morbidity and mortality due to snakebite. Hospital data did not reflect complete scenario of snakebite epidemiology as many people visit traditional healers rather than hospitals for treating snakebite. Such traditional practices might not be effective in snakebite treatment leading to loss of life when bite is from venomous snake. Sensitizing people about the danger of delayed treatment, adopting preventive measures in outdoor or agricultural activities and educating people about the behavior and ecology of snakes might help in reducing the snakebite burden. Sikkim, being located in a geographically difficult position with isolated valleys and continuous torrential rain during monsoon, the transport becomes very challenging during medical emergency. Improving medical infrastructures along with transport facility in every health centers especially in the high risk areas might help in minimizing the burden. Studies should be carried out to establish the regional venom composition so that effective and specific anti-venin can be developed. Development of user friendly application portal for uploading the snakebite cases might be helpful in understanding the epidemiology and, hence formulating the necessary management practices. Finally, involvement of citizen science in studying snakebite epidemiology should be encouraged.

## Supporting information

S1 Text Questionnaire used for interviewing community for collection of data on snake bites cases. (DOCX)

S1 Table Habitat and micro-habitat details of five common venomous snakes of Sikkim. (DOCX)
