# Supplementary material for: Epidemiological study of snakebite cases in Sikkim: Risk modeling with regard to the habitat suitability of common venomous snakes
Source: PLoS Negl Trop Dis. 2021 Nov 8;15(11):e0009800. doi: 10.1371/journal.pntd.0009800 (PMC8601622; doi:10.1371/journal.pntd.0009800)
Supplement: S1 Text — (DOCX) [file pntd.0009800.s001.docx]

**Supplementary File I:** Questionnaire used for interviewing community for collection of data on snake bites cases

**SNAKE BITE CASES**

**DEPARTMENT OF ZOOLOGY**

**SIKKIM UNIVERSITY**

**Name**_____________________________________**Address**____________________________________

**Age**________ **Gender**__________ **Occupation**_______________________________________________

**Qualification:** **A.** No formal education **B.** Basic primary education (1-8 years) **C.** Secondary education

**D.** Higher secondary **E.** Higher studies (class 12 and above)

1. **Have you ever encountered snake?** **A.** Yes **B.** No
2. **In what kind of habitat have you mostly encountered snake?**
3. Farmland
4. Forest
5. Roads
6. Bush
7. Footpaths
8. Urban drains
9. Under the stone
10. Any other habitat
11. **When do you usually encounter snake?**
12. Day
13. Afternoon
14. Evening
15. Night
16. **In which season have you mostly encountered snake?**
17. Pre-monsoon C. Post Monsoon
18. Monsoon (Rainy Season) D. Winter
19. **Have you ever experienced snake bite personally?** A. Yes B. No
20. **Where do you prefer to go?**
21. Hospital
22. Traditional Healer, Why?
23. Quick healing
24. No medical facility in the village
25. Any other reasons________________________________________________________
26. **Time of snakebite:**
27. Day
28. Afternoon
29. Evening
30. Night
31. **Have you heard of snake bite cases in your community?** A. Yes B. No
32. **How many?** ___________________. **Any death cases you know?** _________________________
33. **What is your perception on snakes?**
34. Useless (don’t like) , should be killed whenever/wherever encountered
35. Fear
36. Useful in nature- ecological importance
37. Neutral feeling
38. Any other perception

**Remarks**

______________________________________________________________________________
