# Supplementary material for: Epidemiological study of snakebite cases in Sikkim: Risk modeling with regard to the habitat suitability of common venomous snakes
Source: PLoS Negl Trop Dis. 2021 Nov 8;15(11):e0009800. doi: 10.1371/journal.pntd.0009800 (PMC8601622; doi:10.1371/journal.pntd.0009800)
Supplement: S1 Table — (DOCX) [file pntd.0009800.s002.docx]

**S1 Table:** Habitat and micro-habitat details of five common venomous snakes of Sikkim

| **Species** | **Site** | **Coordinates** | **Elevation**  **(m)** | **Micro-habitat** | **Habitat** | **Distance from human habitation** |
| --- | --- | --- | --- | --- | --- | --- |
| *Ovophis monticola* | Yuksom, West Sikkim | 27° 23' 47.7" N  88° 13' 17.2" E | 2100 | Under leaf litter in cardamom plantation | Temperate broadleaf forest | 500m |
|  | Bulbuley, East Sikkim | 27° 22' 3.23" N  88° 37' 46.86" E | 2094 | Along the bamboo thickets | Temperate broadleaf forest | 2000m |
|  | Ralong, South Sikkim | 27° 19' 41.83" N  88° 21' 1.46" E | 2225 | Inside herbs such as *Arisaema* sp., *Persicaria nepalensis*, Rumex sp. | Temperate broadleaf forest | 100m |
|  | Chungthang, North Sikkim | 27° 36' 17.66" N  88° 38' 48.5" E | 1617 | Under leaf litter in cardamom plantation | Temperate broadleaf forest | 200m |
|  | Theng, North Sikkim | 27° 34' 56.05" N  88° 39' 12.58" E | 1649 | Inside herbs in agriculture farm | Temperate agro- forest | 300m |
|  | Selep Busty, East Sikkim | 27° 18' 16.04" N  88° 21' 9.46" E | 1740 | Road killed | Temperate broadleaf forest | 800m |
|  | Barfung Block  South Sikkim | 27° 17' 42.57" N  88° 21' 9.16" E | 1839 | Maize field | Temperate agro- forest | 30m |
|  | Ravangla,  South Sikkim | 27° 18' 31.04" N  88° 21' 37.22" E | 2009 | Inside herbs such as *Arisaema* sp., *Persicaria nepalensis*, Rumex sp. | Temperate broadleaf forest | 200m |
|  | Bhusuk,  East Sikkim | 27° 21' 3.54" N  88° 40' 22.15" E | 1630 | Near the wooden bridge. | Temperate agro-forest | 150m |
|  | Singhik, North Sikkim | 27° 31' 4.94" N  88° 33' 11.75" E | 1285 | In drains along the village road | Mixed forest with bamboo patches | 500m |
|  | Regu, East Sikkim | 27° 11' 38.27" N  88° 42' 7.51" E | 1440 | Agriculture field | Tropical agro-forest | 300m |
|  | Soreng,  West Sikkim | 27° 9' 56.72" N  88° 12' 1.09" E | 1492 | In cardamom plantation | Temperate broadleaf forest | 300m |
|  | Chalamthang, South Sikkim | 27° 12' 55.89" N  88° 28' 20.7" E | 837 | Village trail with leaf litters. | Tropical agro-forest | 500m |
|  | Uttaray,  West Sikkim | 27° 15' 56.02" N  88° 5' 43.8" E | 2100 | Agriculture field | Temperate forest dominated by Oak trees | 50m |
|  | Central Pandam,  East Sikkim | 27° 12' 12.96" N  88° 33' 49.95" E | 857 | Inside the broom grass (*Thysanolaena* sp.) | Tropical agro-forest | 100m |
| *Protobothrops himalayanus* | Chungthang,  North Sikkim | 27° 36' 17.66" N  88° 38' 48.5" E | 1620 | Moist litter of cardamom plantation | Temperate agro-forest | 300m |
|  | Bob,  North Sikkim | 27° 37' 4.79" N  88° 39' 35.59" E | 1750 | Within the tall grasses of agricultural fields | Temperate agro-forest | 100m |
|  | Theng,  North Sikkim | 27° 34' 56.05" N  88° 39' 12.58" E | 1650 | Road side drain | Tropical broadleaf forest | 800m |
|  | Bitchu,  North Sikkim | 27˚ 41' 13" N  88˚ 44' 40" E | 2340 | Within the grasses in pastoral land | Pastoral land surrounded by temperate forest | 300m |
|  | Toong,  North Sikkim | 27° 33' 6.17" N  88° 38' 48.49" E | 1460 | Inside the gap between two rocks | Temperate agro-forest | 500m |
|  | Khedum,  North Sikkim | 27° 37' 32" N  88° 41' 35.8" E | 2050 | Along the cervix of huge stone. | Pastoral land surrounded by temperate forest | 300m |
|  | Maltin,  North Sikkim | 27° 37' 34.59" N  88° 40' 21.8" E | 1982 | Near the power office | Temperate broadleaf forest | 500m |
|  | Pegong,  North Sikkim | 27° 34' 57.2" N  88° 39' 13.43" E | 1643 | Road killed | Temperate broadleaf forest | 200m |
| *Bungarus niger* | Lingmoo,  South Sikkim | 27° 19' 39.5" N  88° 27' 53.3" E | 975 | Dead and slightly dried specimen along the village trail | Tropical agro-forest | 200m |
|  | 6th Mile, Gangtok, East Sikkim | 27° 18' 16.03" N  88° 35' 17.02" E | 1006 | Footpath connecting village with highway | Human habitation | - |
|  | 7th mile Gangtok, East Sikkim | 27° 18' 0.39" N  88° 35' 19.76" E | 976 | Basement of house | Human habitation | - |
|  | Sirwani,  South Sikkim | 27° 14' 27.4" N  88° 28' 39.48" E | 380 | Dead specimen inside drain along the road | Tropical agro-forest | 200m |
|  | Dalep,  South Sikkim | 27° 14' 32.1" N  88° 28' 4.9" E | 616 | Near the bridge | Tropical forest dominated by *Schima wallichi* and *Shorea robusta* | 500m |
|  | Bermoik,  South Sikkim | 27° 13' 53.07" N  88° 28' 32.14" E | 871 | Dead specimen in the village trail | Tropical agro-forest | 50m |
|  | Kitam,  South Sikkim | 27° 6' 21.18" N  88° 21' 22.2" E | 498 | Village roadside | Tropical forests dominated by *Shorea robusta* and *Pinus* sp. | 100m |
|  | Metro, Gangtok, East Sikkim | 27° 18' 42.66" N  88° 35' 24.2" E | 1107 | Inside the drain of village footpath | Human habitation | 100m |
|  | Ranka,  East Sikkim | 27° 20' 31.74" N  88° 35' 8.24" E | 1250 | Village roadside | Human habitation | - |
| *Naja kaouthia* | Lingee,  South Sikkim | 27° 22' 27.88" N  88° 28' 2.7" E | 1050 | Near the cracked walls of toilet | Human habitation | - |
|  | Dalep,  South Sikkim | 27° 14' 32.1" N  88° 28' 4.9" E | 450 | Inside chicken’s house | Human habitation | - |
|  | Legship,  West Sikkim | 27° 16' 51.04" N  88° 16' 32.11" E | 584 | Near the cemented water tank of village | Tropical agro-forest | 100m |
|  | Pacheykhani,  East Sikkim | 27° 12' 40.36" N  88° 36' 10.39" E | 1020 | Near the stream | Tropical agro-forest | 500m |
|  | Kwezing,  West Sikkim | 27° 18' 18.16" N  88° 13' 30.08" E | 1500 | Agriculture farm | Tropical agro-forest |  |
|  | Rongli Bazar,  East Sikkim | 27° 12' 12.13" N  88° 42' 5.05" E | 826 | In the market area near shop | Human habitation | - |
|  | Ralang,  South Sikkim | 27° 19' 31.6" N  88° 19' 27.2" E | 1805 | Along the stone wall of Ralang old monastery | Human habitation | - |
|  | Ranipool,  East Sikkim | 27° 17' 26.3" N  88° 35' 36.26" E | 851 | On the play ground of school. | Human habitation | - |
|  | Pangthang,  East Sikkim | 27° 22' 37.4" N  88° 37' 18.16" E | 1655 | In the park at Golitar | Temperate broadleaf forest | 3000m |
|  | Tathangchen,  East Sikkim | 27° 20' 34.87" N  88° 38' 51.48" E | 1740 | Near bamboo thickets | Human habitation | - |
|  | Tharpu,  West Sikkim | 27° 8' 33.5" N  88° 11' 26.06" E | 876 | Near the streams | Tropical riverine forest | 400m |
| *Ophiophagus hannah* | Rangpoo,  East Sikkim | 27° 10' 34.66" N  88° 31' 40.03" E | 300 | On village footpath | Tropical agro-forest | 300m |
|  | Ravangla,  South Sikkim | 27° 18' 22.88" N  88° 21' 50.79" E | 2009 | In front of the house | Human habitation | - |
|  | Selep Busty,  East Sikkim | 27° 18' 12.47" N  88° 21' 10.01" E | 1740 | Near the spring water | Temperate broadleaf forest | 300m |
|  | Central Pandam  East Sikkkim | 27° 12' 16.98" N  88° 33' 48.03" E | 857 | Inside paddy field | Agriculture farm | 100m |
|  | Yuksom,  West Sikkim | 27° 22' 16.6" N  88° 13' 12.83" E | 1820 | Inside the drain adjoining human settlement | Temperate broadleaf forest | 200m |
|  | Sadam,  South Sikkim | 27° 7' 3.48" N  88° 25' 31.6" E | 1360 | Inside agricultural field | Mixed farming where cabbage, cauliflower and carrot were grown. | 80m |
|  | Kitam,  South Sikkim | 27° 7' 7.23" N  88° 21' 6.46" E | 550 | Inside dry leaf litters | Tropical forest dominated by *Shorea robusta* | 1000m |
|  | Legship,  West Sikkim | 27° 16' 47.08" N  88° 16' 29.51" E | 560 | Along the roadside drain | Tropical forest | 20m |
|  | Ralang,  South Sikkim | 27° 19' 48.3" N  88° 20' 8.81" E | 1850 | Near water tap outside the house | Human habitation | - |
